# Atlas of Skin Diseases

**Published:** 1876-08

**Authors:** 


### Books Reviewed.

*Atlas of Skin Diseases. Part I, Eczema (Erythematosum) ,Psoriasis, Lupus Erythematosus, Syphiloderma (pustulosum).* By Louis A. Duhring, M. D., Professor of Skin Diseases in the University of Pennsylvania. Philadelphia: J. B. Lippincott & Co., 1876. Buffalo: T. Butler & Son.

The appearance of an American Atlas of Skin Diseases is something, we believe, entirely new in the history of American literature. There is no doubt that the difference in climate, food and customs of this country from the Old World, produces a marked variation in the appearance of the diseases observed here from those seen in Europe, and for this reason the illustrations of skin disease, as seen in the Atlas of Hebra for instance, are not such as would enable a practitioner who had not received special advantages in that line, to recognize the same disease in America. It is a fact which will commend this Atlas to an American reader, that while at the first glance he may be disappointed for the reason that it does not represent the diseases as classically described, he will find that the illustrations are true to life, and represent the ordinary American types of skin diseases.

The illustrations are accompanied by descriptive text which is simple and to the point, the treatment used in each case figured, which are all taken from actual practice, is also given. Dr. Duhring is to be congratulated upon the fine appearance of the initial number of his Atlas. From the high reputation of the author, as a clinical teacher and writer, it can safely be said that the subsequent numbers will full bear out the promise of excellence here put forth.

